# En bloc radical cystectomy: An overview of the technique and oncological results

**DOI:** 10.1002/bco2.190

**Published:** 2022-09-18

**Authors:** Eirik Kjøbli, Øyvind Salvesen, Sverre Langørgen, Øystein Størkersen, Arne Wibe, Carl‐Jørgen Arum

**Affiliations:** ^1^ Department of Clinical and Molecular Medicine Norwegian University of Science and Technology (NTNU) Trondheim Norway; ^2^ Department of Surgery, St. Olavs Hospital Trondheim University Hospital Trondheim Norway; ^3^ Department of Radiology and Nuclear Medicine, St. Olavs Hospital Trondheim University Hospital Trondheim Norway; ^4^ Department of Pathology, St. Olavs Hospital Trondheim University Hospital Trondheim Norway

**Keywords:** bladder cancer, cystectomy, en bloc, local recurrence, lymph node dissection, lymph node excision, oncologic results, urinary bladder neoplasms, urothelial cancer

## Abstract

**Objectives:**

To reduce recurrence after radical cystectomy (RC), we developed a technique based on the principles of the circumferential resection margin used during total mesorectal excision for rectal cancer, namely, en bloc radical cystectomy (EbRC).

**Patients and methods:**

The study included all patients in Mid‐Norway (population of 739 k) with high‐grade superficial or muscle invasive bladder cancer considered for radical treatment according to European guidelines, from January 2012 to August 2021, except for three patients receiving trimodal therapy. One hundred forty‐five patients were treated with EbRC and 188 patients with standard RC (stdRC). There were no exclusion criteria. Both groups included open and robot‐assisted techniques. EbRC entails cystectomy with extended pelvic lymph node dissection. The technique focuses on systematic uninterrupted mobilisation of all lymphatic tissue from the circumferential resection margin towards the bladder pedicles, and resecting the tissue en bloc with the bladder.

**Results:**

The 3‐year recurrence‐free survival (RFS) was 86% for EbRC versus 67% for stdRC. The hazard ratio for overall survival in multivariable cox regression analyses after EbRC versus stdRC was 0.30 (95% CI 0.16–0.57, *p* ≤ 0.001). The improved outcomes persisted in propensity score‐matched analyses. There were no differences in Clavien–Dindo 3 and 4 complications (12.4% vs. 11.7%), nor 90‐day mortality (2.1% vs. 1.6%).

**Conclusion:**

Improved oncological results with EbRC versus stdRC mirror the historical data after total mesorectal excision was initiated over 35 years ago in rectal cancer surgery. EbRC is safe and the preliminary oncological results are promising.

## 1 INTRODUCTION

Reported local recurrence (LR) rates after RC with lymph node dissection for high grade superficial or muscle invasive bladder cancer are from 6% to 54%,^1^, ^2^, ^3^ and 5‐year RFS rates vary from 59% to 69%.^4^, ^5^, ^6^, ^7^ Rink et al. published a median cancer specific survival after recurrence of 6.9 months, and 83% of patients with recurrence died from the disease within 2 years.^8^

**FIGURE 1:**
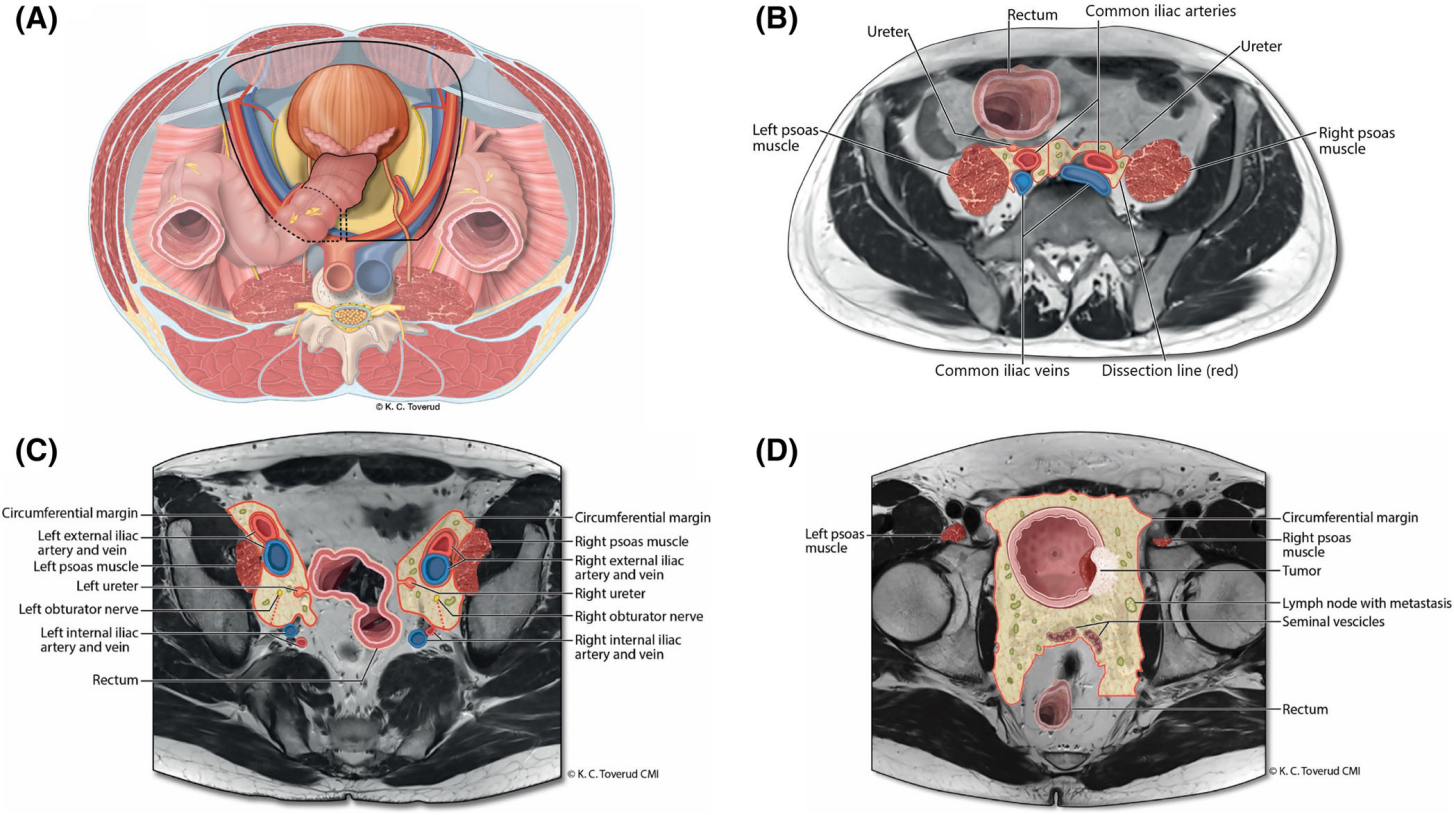
(A) Overview of the pelvis. The black line represents the circumferential resection margin performing en bloc Radical Cystectomy. The genitofemoral nerve is the lateral border, the aortic bifurcation the cranial border, the rectum the medial border and the dissection is executed down to the branches of the internal iliac vessels and endopelvic fascia. (B) MR‐image at the level of the common iliac vessels. (C) MR‐image at the level of obtorator fossa. (D) MR‐image at the level of the bladder. The bladder is illustrated with a tumour on the right side and a lymph node with metastasis. Central anatomical structures in proximity to the dissection are illustrated in image B–D. The red lines represents the circumferential resection margin.

To reduce recurrence, we developed a surgical approach for RC with en bloc resection of the bladder and all its associated lymphatic tissue (termed EbRC; Figure 1A). The technique entails extended pelvic lymph node dissection as described in the European guidelines (Figure 1A–D).^1^

EbRC adheres to the same surgical principles as total mesorectal excision (TME), first described by Heald in 1986.^9^ Implementing TME as standard technique for surgical treatment of rectal cancer significantly reduced LR rates from 30% to 5% and increased 5‐year survival rates from 50% to 75%.^10^ The causes of LR are as follows: The proximity of the tumour and lymph node metastasis (pN+) to the circumferential margin, extranodal extension, extramural venous invasion, tumour differentiation, lymphovascular invasion, perineural infiltration, carcinoma in situ (CIS), variant histology, tumour cell seeding, and residual lymph nodes with micrometastases. Neoadjuvant chemotherapy certainly reduces the above‐mentioned phenomena, but not completely.^11^ Recurrence after RC may to some degree reflect a surgical technique that does not adequately address anatomical, oncological, or embryonic principles.^1^, ^2^, ^3^

The embryonic development of the lymphatic as well as venous drainage of the urinary bladder and rectum are morphologically similar.^12^ Initiating the procedure with dissection at the outer anatomical borders of the pelvic lymphatic drainage of the bladder and removing the entire specimen en bloc could potentially decrease the risk of positive surgical margins, vascular seeding, and incomplete removal of extravesical viable tumour tissue.^13^, ^14^ In this manner, the embryonic space of the urinary bladder is removed en bloc (Figure 1A–D). This encompasses uninterrupted excision of the soft tissue margin to the tumour‐affected organ as well as lymph node metastases, and all regional lymphovascular structures. Addressing the circumferential resection margin is the main principle of both TME and EbRC.

Guidelines in other specialties of oncological surgery emphasise the removal of lymph nodes and tumour en bloc.^14^, ^15^, ^16^ Lymph node dissection at RC has traditionally been performed separately; however, the rationale behind separate excision of lymph nodes and the bladder specimen has not been questioned. There is an ongoing discussion as to the upper boundary of the lymph node dissection: standard, extended, or super‐extended.^17^ There is one randomised study on this topic showing no significant survival differences between extended and standard lymph node dissection.^5^ Authors of several nonrandomised studies have presented data suggesting improved results for extended versus standard lymph node dissection.^18^ Although in numerous publications researchers have regarded the controversial upper limit, no authors have discussed the en bloc surgical approach.

The present study was conducted to evaluate the preliminary results of EbRC versus standard radical cystectomy, reporting on recurrence, survival, and complication rates.

## 2 PATIENTS AND METHODS

### 2.1 Study population

Data were collected on 343 consecutively treated patients with RC at our department (tertiary treatment centre of Mid‐Norway, population 739 k) from January 2012 to August 2021. All patients considered for radical treatment were included, except for three patients who were treated with trimodal therapy. A total of 192 patients were treated with stdRC from January 2012 to August 2018, and 151 patients underwent EbRC from January 2017 to August 2021. The indication for cystectomy was high‐grade nonmuscle invasive or muscle invasive bladder cancer according to the European Association of Urology (EAU) Guidelines.^1^ There were no exclusion criteria. Patients with prior radiation therapy or prostatectomy were also included and represent <5% of the study population. The preoperative workup included cystoscopy, computed tomography (CT) of the thorax/abdomen/pelvis, blood tests, transurethral resection of the bladder, clinical examination, and evaluation by a multidisciplinary team. Radiological and pathological evaluations were performed by the same uroradiologists and uropathologists for all patients. The pathological stages (pT) were defined according to the 8th edition of the Union for International Cancer Control (UICC). Neoadjuvant chemotherapy (dose‐dense methotrexate, vinblastine, doxorubicin, and cisplatin) and evaluation by a multidisciplinary team were implemented at our department as of January 2012. Despite full preoperative work‐up, not all findings suspicious of metastases could be verified. Six EbRC and four stdRC patients were diagnosed with progressing preoperative findings within 6 months of RC and therefore re‐classified as initial metastatic disease (M1), leaving 145 EbRC patients and 188 stdRC patients for analysis.

To limit confounding factors and possible biases, both populations represent consecutively treated patients, operated on by the same two surgeons. Postoperative follow‐up schedules were identical, all patients were from the same geographic region, and all study investigators had full access to journals at referring and follow‐up hospitals. During follow up, only three patients were lost due to emigration (two stdRC patients after 2 and 4 years and one EbRC patients after 6 months). All three are still alive.

The senior surgeon had performed >300 RC as of 2012. The surgeon performing EbRC had 4 years of experience with RC and had performed 600 robot‐assisted radical prostatectomies (RARP). The surgeon had trained in removing the lymph node specimen en bloc while performing RARP, so there were only minor challenges in evolving stdRC to EbRC.

All patients in both groups were followed according to European and national guidelines for bladder cancer (Norwegian Directorate of Health^19^). The follow up schedule included blood tests and clinical examination every 6 months for 5 years, and CT scan (thorax/abdomen/pelvis) every 6 months for the first 2 years and annually for the next 3 years. All hospitals had high quality digitalized imaging labs in the study period.

LR was defined as recurrence within the confines of the pelvis, below the aortic bifurcation, in the region defined by extended lymph node dissection, including the surgical site of the cystectomy, carcinomatosis, and recurrence in the ureteroileal anastomosis. All LRs were registered, including LR occurring in patients with distant metastases. All LRs were confirmed by a dedicated uroradiologist.

The study was approved by the Regional Ethics Committee, reference number 2019/236.

### 2.2 Surgical technique

EbRC is completed within the anatomical landmarks of stdRC, with similar steps in a specific order, resulting in an en bloc technique. Both techniques can be performed with standard or extended lymph node dissection. We emphasise adhering to the principles of non‐touch technique.^13^ The accuracy of modern imaging has made manipulation to evaluate resectability less relevant. The surgical approach is identical in both open and robot‐assisted surgery.

Mobilise the rectosigmoid colon for visualisation and access to the left iliac vessels and ureter. Incise peritoneum lateral to the external iliac vessels. Dissect along the genitofemoral nerve to the inferior epigastric vessels and mobilise all lymphatic tissue medially to the external iliac vessels. Continue the dissection towards the internal opening of the inguinal canal and expose Cooper's ligament. Identify the entrance to the obturator foramen and obturator nerve. Isolate the ureter with atraumatic technique to the level of the bladder wall, clip, and divide. Mobilise all lymphatic tissue anterior to the common iliac vessels medially towards the presacral plane (Figure 1B) up to the aortic bifurcation. Access the space dorsal to the hypogastric nerve, divide the specimen in the midline anterior to the sacrum, and lateralise it towards the bladder pedicle. Retract the external iliac vessels medially and enter the fossa of Marseille. Identify and isolate the obturator nerve and vessels. Mobilise all lymphatic tissue down to and between the branches of the internal iliac vessels towards the bladder pedicle (Figure 1C). Perform a similar dissection on the right side and continue with incision of the peritoneum anterior to the rectum. Identify the seminal vesicles and expose the space between the prostate and rectum. Dissect the bladder pedicles from the internal iliac vessels down to the prostate pedicles or vagina. Assist the dissection in females with vaginal sponges and continue the dissection either between the bladder and anterior vaginal wall or bilaterally through the anterolateral vaginal wall towards the urethra. Preserve the neurovascular bundles in accordance with tumour stage.

**FIGURE 2:**
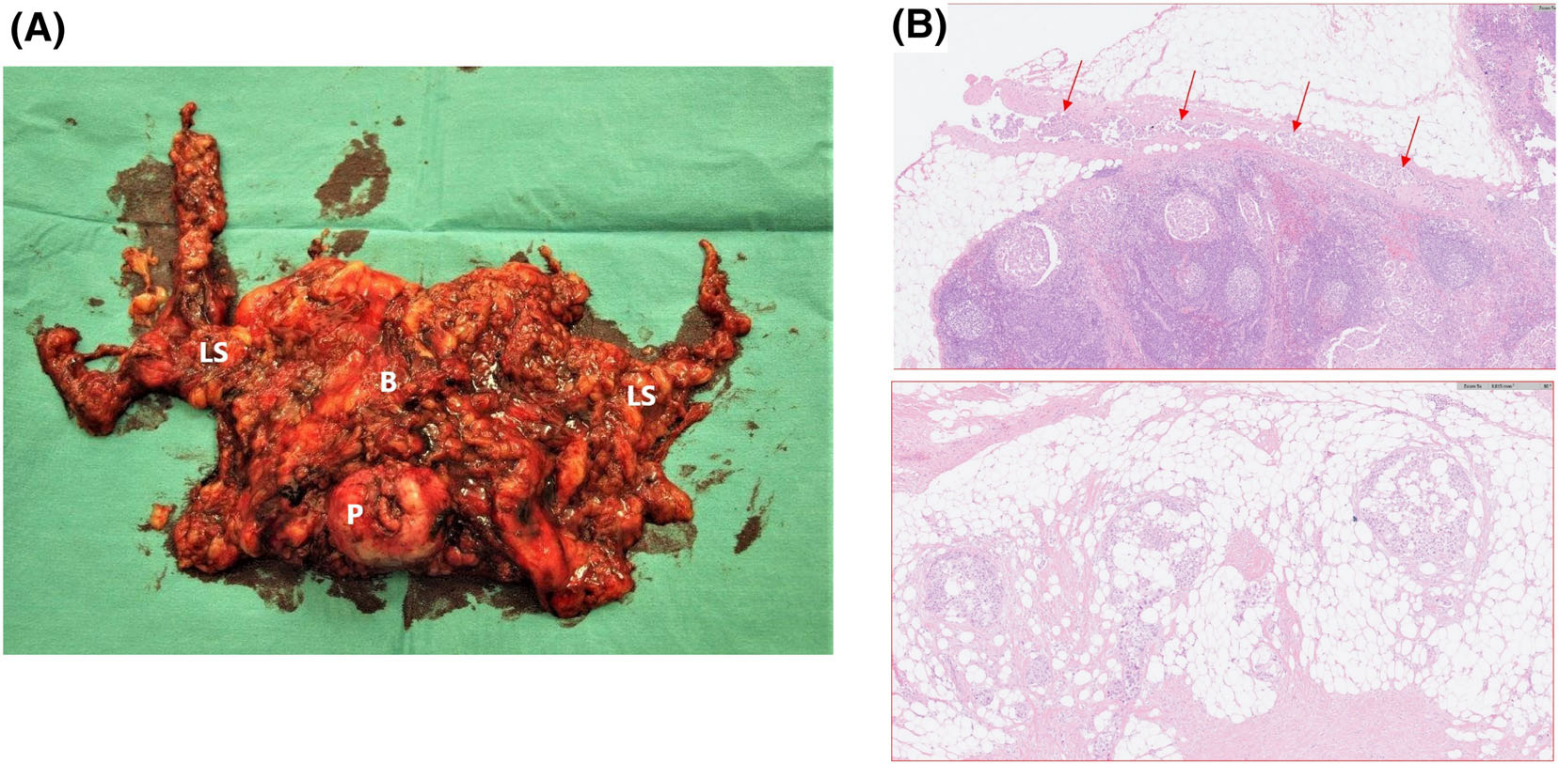
(A) Specimen after En bloc Radical Cystectomy. P: prostate, B: bladder, and LS: lymph node specimen. (B) Histopathological images (haematoxylin and eosin [H&E] stain). Both images are from the pelvic lymph node dissection specimen. The upper image shows cancer cells in lymphatic vessels (arrows) outside the lymph node. The lower image shows remaining cancer cells in fatty tissue in the proximity to a lymph node metastasis after neodjuvant chemotherapy. Both findings represent an increased risk of recurrence if treated with stdRC compared with EbRC.

Dissection varies according to prior surgery or radiation therapy. Our experience is that also in these cases one can dissect within the lateral borders with the en bloc technique. Mobilise the bladder from the anterior pelvic wall and symphysis pubis, and ligate Santorini's plexus. Remove the catheter and then clip and divide the urethra. The specimen is shown in Figure 2A.

### 2.3 Statistical analysis

Patient characteristics at surgery for the two treatment groups are summarised by using the median for continuous variables and proportions for categorical variables. Differences between the groups were examined by using the Mann–Whitney *U* test for continuous variables and Fisher's exact test for categorical variables. Local recurrence‐free survival (LRFS), RFS, cancer specific survival (CSS), and overall survival (OS) in the subpopulations M0 patients and M0/clinical stage (cT) ≥ 2 patients were estimated with Kaplan–Meier analysis. For all combinations of events and subpopulations, the treatment groups were compared using the log‐rank test. Multivariable Cox regression analyses were performed for LRFS, RFS, CSS, and OS in M0 patients adjusting for age, gender, neoadjuvant chemotherapy, Charlson comorbidity index (CCI), lymph node metastases at diagnoses, CIS, pathological stage (final pathology report), and pTNM‐stage (UICC). *p* values <0.05 were considered statistically significant. Propensity score matching was performed with the variables adjusted for in the multivariable Cox regression analyses. Kaplan–Meier analyses were conducted on the propensity score–matched data. Statistical analyses were done by using R version 3.6.3 and SPSS version 27.0.1.0.

## 3 RESULTS

No significant differences were found comparing the two groups for gender, age, comorbidity, neoadjuvant chemotherapy, or clinical stages (Table 1). The patients were chosen for open or robot‐assisted technique based on availability of the robot and the presence of prior major abdominal surgery. All urinary reconstructions in robot‐assisted procedures were performed intracorporeally.

**TABLE 1:** Baseline patient characteristics

Baseline characteristics	EBRC (*N* = 145)		Standard (*N* = 188)		*p* value
Age (years)	70.3	(63–77)	70.8	(64–77)	0.4
Men	113	(78%)	146	(78%)	
CCI	5	4‐6	5	4‐6	0.3
Neoadjuvant chemotherapy (cT ≥ 2)	52/104	(50%)	64/142	(45%)	0.5
Clinical and TURBT stage					0.9
TIS	2	(1%)	1	(0.5%)	
T1	39	(27%)	45	(24%)	
T2	62	(43%)	86	(46%)	
T3	32	(22%)	41	(22%)	
T4A	9	(6%)	14	(7%)	
T4B	1	(1%)	1	(0.5%)	
Histologic type					0.7
Urothelial carcinoma	133	(92%)	177	(94%)	
Squamous cell carcinoma	3	(2%)	2	(1%)	
Adenocarcinoma	2	(1%)	3	(2%)	
Small cell carcinoma	4	(3%)	5	(3%)	
Sarcoma	3	(2%)	1	(0.5%)	
cN1‐2	10	(7%)	22	(12%)	0.19
CIS	76	(53%)	69	(37%)	0.005
WHO‐GRADE 3	138	(96%)	179	(96%)	1

*Note*: Patient characteristics at surgery for the two treatment groups summarised using the median (Q1, Q3) for continuous variables and *n* (%) for categorical variables. Differences between the groups are examined using the Mann–Whitney *U* test for continuous variables and Fisher's exact test for categorical variables. CCI: Charlson Comorbidity Index. cN: clinical lymph node stage before surgery, based on CT‐scan and/or MRI. CIS: carcinoma in situ. WHO‐grade: World Health Organization grades (1–3), 3: poorly differentiated.

Eighty‐eight per cent of EbRC and 15% of stdRC were performed with robot‐assisted surgery. Extended lymph node dissection was performed in 58% (84/145) of the EbRC patients and in 17% (32/188) of the stdRC patients. The median time of surgery was 219 min for EbRC and 230 min for stdRC. The stdRC group had a significantly higher blood loss and transfusion rate compared with the EbRC group (700 vs. 300 ml, *p* < 0.001).

Clavien–Dindo grade 2 complications were noted in 32% in the EbRC group versus 47% in the stdRC group (*p* < 0.005). Grade 3–4 complications were noted in 12.4% versus 11.7% in the same two groups, respectively (Table 2). The 90‐day readmission rate was 19% in the EbRC group and 15% in the stdRC group (*p* = 0.38), and the 90‐day mortality rate was 2.1% versus 1.6% (*p* = 1).

**TABLE 2:** Perioperative and pathological outcomes

Perioperative and pathological outcomes	EbRC (*N* = 145)		Standard (*N* = 188)		*p* value
OR‐time (min)	219	(191–264)	230	(203–285)	0.018
Blood loss (ml)	300	(150–500)	700	(450–1170)	<0.001
Follow up (months)	20	(10–39)	50	(19–73)	
Robotic surgery	127/145	(88%)	29/188	(15%)	<0.001
PT (pathology report after cystectomy)^a^					0.015
T0	50	(35%)	56	(30%)	
Tis	5	(3%)	0	(0%)	
T1	25	(17%)	20	(11%)	
T2	18	(12%)	40	(21%)	
T3	35	(24%)	50	(27%)	
T4A	12	(8%)	21	(11%)	
T4B	0	(0%)	1	(0.5%)	
Lymph nodes in specimen	16	12–22	12	7–17	
Standard PLND	14	(9–18/*n* = 61)	11	(7–15/*n* = 138)	0.029
Extended PLND	19	(13–24/*n* = 84)	17	(12–25/*n* = 32)	0.4
pT–TNM (UICC)^a^					0.5
Tis	2	(1%)	0	(0%)	
T1	35	(24%)	35	(19%)	
T2	45	(31%)	59	(31%)	
T3	48	(33%)	69	(37%)	
T4A	14	(10%)	23	(12%)	
T4B	1	(1%)	2	(1%)	
pN+^b^	17	(12%)	42	(22%)	0.014
Local recurrence	7	(4.8%)	39	(20.7%)	
Local recurrence/pN+	1/17	(5.9%)	19/42	(45%)	0.005
Positive surgical margins	0	(0%)	13	(7%)	<0.001
Metastasis					
Lung	3	(2.1%)	20	(11%)	0.002
Liver	2	(1.4%)	23	(12%)	<0.001
Skeleton	3	(2.1%)	28	(15%)	<0.001
Brain	2	(1.4%)	5	(2.7%)	0.7
Port site	0	(0%)	0	(0%)	
Carcinomatosis	2	(1.4%)	10	(5.3%)	0.075
Distant lymph node	6	(4.1%)	27	(14%)	0.003
Multivariable cox regression analyses:					
Local recurrence free survival:					
	HR:	95% CI	*p*		
Unadjusted EBRC vs. stdRC	0.26	0.12–0.59	≤0.001		
Adjusted EBRC vs. STRC	0.29	0.13–0.65	0.003		
Recurrence free survival:					
	HR:	95% CI	*p*		
Unadjusted EBRC vs. stdRC	0.32	0.18–0.58	≤0.001		
Adjusted EBRC vs. stdRC	0.36	0.20–0.64	≤0.001		
Cancer specific survival:					
	HR:	95% CI	*p*		
Unadjusted EBRC vs. stdRC	0.25	0.12–0.53	≤0.001		
Adjusted EBRC vs. stdRC	0.28	0.13–0.60	0.001		
Overall survival					
	HR:	95% CI	*p*		
Unadjusted EBRC vs. stdRC	0.28	0.15–0.52	≤0.001		
Adjusted EBRC vs. stdRC	0.30	0.16–0.57	≤0.001		

*Note*: Patient characteristics after surgery and during follow up. Findings for the two treatment groups summarised using the median (Q1, Q3) for continuous variables and *n* (%) for categorical variables. Differences between the groups were examined using the Mann–Whitney *U* test for continuous variables and the Fisher's exact test for categorical variables. OR: operating room. pN+: lymph node metastases in final pathology report. Multivariable cox regression analysis performed for local recurrence free survival, recurrence free survival, cancer specific survival and overall survival adjusting for age, gender, neoadjuvant chemotherapy, Charlson comorbidity Index, lymph node metastases at diagnoses, carcinoma in situ, pT: pathological stage (final pathology report) and pTNM‐stage (Union for International Cancer Control, eight edition). EbRC: en bloc radical cystectomy. stdRC: standard radical cystectomy. HR: hazard ratio. CI: confidence interval.

^a^ Staging according to the 8th edition of The Union for International Cancer Control (UICC).

^b^ Pathological examination for en bloc dissection does not enable differentiation between groups N1 to N3.

LR rates after stdRC was 22% for surgeon 1 and 18% for surgeon 2 (*p* = 0.9), and 43% versus 46% respectively for patients with pT ≥ 3 (*p* = 1). LR was diagnosed in 7/145 of patients in the EbRC group and 39/188 of patients in the stdRC group (Figure 2A, *p* < 0.001). For pN+ patients, the LR rate was 1/17 (5.9%) in the EbRC group and 19/42 (45%) in the stdRC group (*p* < 0.005). Positive surgical margins were found in 7% (13/188) of the patients in the stdRC group and in none of the patients in the EbRC group. In the EbRC group, four out of seven LRs were found in patients treated during the first year developing the technique and two out of seven in the second year. Four out of seven LRs were found within 7 months.

**FIGURE 3:**
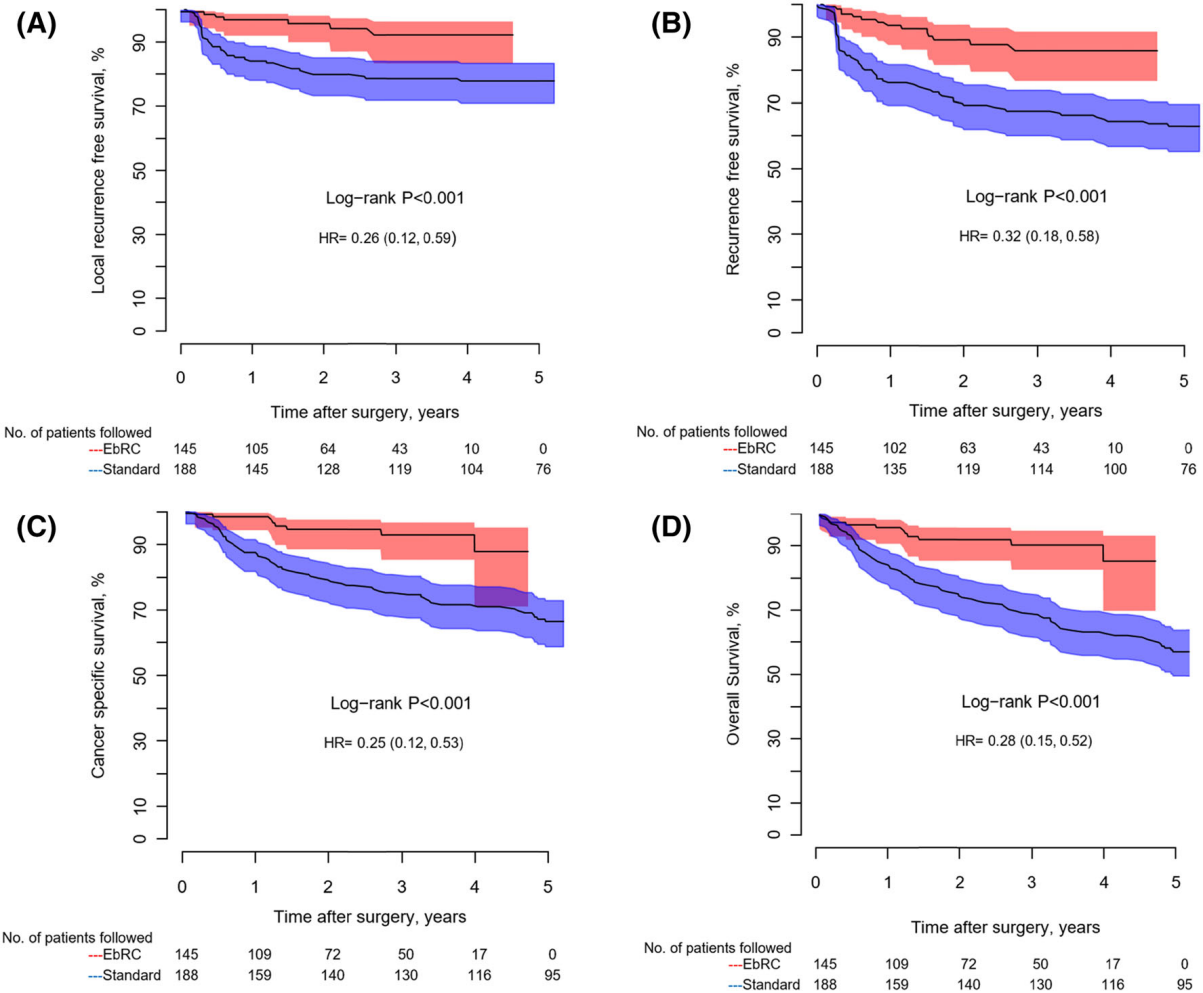
Kaplan–Meier curves comparing (A) local recurrence‐free survival, (B) recurrence‐free survival, (C) cancer specific survival, and (D) overall survival, for all patients in both groups. The number of patients followed without an event in each group are reported annually.

Metastases were diagnosed during follow up in 14/145 of the EbRC patients and 65/188 of the stdRC patients (*p* < 0.001). The sites and incidence of metastases in both groups are presented in Table 2. Improved oncological outcomes for the EbRC group were noted by comparing Kaplan–Meier analyses displaying the incidence of LRFS, RFS, CSS, and OS (Figures 3A–D). Improved outcomes persisted when excluding ≤T1 disease. Outcomes for clinical stage ≥cT2 are presented in Supporting Information S1. During follow up, eight out of 145 in the EbRC group and 59/188 in the stdRC group died from bladder cancer.

Multivariable Cox regression analyses adjusting for age, gender, neoadjuvant chemotherapy, CCI, lymph node metastases at diagnoses, CIS, pathological stage (final pathology report), and pTNM‐stage (UICC) resulted in a hazard ratio for OS of 0.30 (95% confidence interval [CI] 0.16–0.57) and *p* < 0.001 (Table 2; the complete analyses are presented in Supporting Information S2). Significant improved oncological outcomes for the EbRC‐patients persisted throughout analyses with propensity score–matched data. The Kaplan–Meier analyses are presented in Supporting Information S3.

## 4 DISCUSSION

A significant number of patients undergoing RC with the intention to cure experience recurrence. Adding lymph node dissection and neoadjuvant chemotherapy to RC improves the 5‐year OS rate, but it is still in the range of 50%–66%.^5^, ^20^ Despite these changes in treatment, recurrence and survival rates after RC have only marginally improved over the last decades.^21^

To improve the surgical technique, we reviewed cancer treatment of organs with the same embryonic origin. Improved oncological results after implementing TME (35 years ago) for rectal cancer surgery represented a paradigm shift. Colorectal surgeons directed their focus towards reducing the risk of losing malignant cells from the tumour‐associated venous and lymphovascular tissues. The same risk factors account for recurrence after bladder cancer surgery.^16^, ^22^, ^23^, ^24^ To achieve TME‐like surgery at RC, the essence of EbRC is the complete excision of the entire lymphatic drainage of the bladder, including all tissue starting at the outer landmarks of the resection (Figure 1A–D) without interrupting lymphovascular structures. By applying the *circumferential resection margin* concept to RC, one increases both tumour and nodal margins and thereby decreases the risk of lymphovascular and venous seeding of cancer cells (Figure 2B). The principle of the *circumferential resection margin* addresses that 50% of lymph node metastases have extranodal extensions that correlate with poor prognosis.^25^ LR for pN+ patients in the EbRC group was 6% (1/17), while it was 45% (19/42) in the stdRC group.

TME and EbRC emphasise early devascularisation of the tumour‐bearing organ and nontouch technique. A nontouch technique is more completely achieved with EbRC compared with stdRC due to the order of the steps in the technique and tissue handling. A reduction in distant metastases has been noted after EbRC (10% vs. 35%), equivalent to the results of TME for rectal cancer.^26^

Elsayed et al.^4^ published an 11% LR rate after robot‐assisted RC compared with 4.8% in the present EbRC group (88% robot‐assisted RC), with a 26‐month versus 20‐month follow‐up. Their patient population had a 15% lower neoadjuvant chemotherapy rate compared with the EbRC group, and a 10% lower incidence of preoperative stages ≥cT3, but equivalent distribution of stages in the final pathology report. To date, no studies have shown significant oncologic superiority for the robot‐assisted technique versus open RC.^27^, ^28^

A 21% local recurrence rate after stdRC might seem high, but it is at the same level (22%) as reported from the Swedish Bladder Cancer Registry by Sabir et al.^29^

Publications on results after RC for bladder cancer^4^, ^5^, ^6^, ^30^ present similar RFS and OS despite the inhomogeneous patient populations regarding stages, surgical approaches, and chemotherapy. These and other authors have reported similar survival as after stdRC at our centre (Table 3). Unchanging outcomes over time despite improvements in surgical devices might reflect the limitations of stdRC. These studies and our stdRC outcomes match the oncological results after rectal cancer surgery before implementing TME for rectal cancer.

**TABLE 3:** Patient population characteristics and outcomes in the EbRC and stdRC group compared with previous publications

	Gschwend et al.		Elsayed et al.	Stein et al.	Madersbacher et al	Steven et al	Mitra et al		Hautmann et al.	Venkatramani et al.		Kjøbli et al.	
	Limited	Extended	Robotic				All	2010–2018		Open	Robotic	stdRC	EbRC
No. patients	203	198	2107	1054	507	336	3347	1145	1100	152	150	188	145
Year of surgery	2006–2010			1971–1997	1985–2000	1993–2005	1971–2018		1986–2009	2011–2014		2012–2018	2017–2021
Gender % male	80%	76%	76%	80%	79%	79%	80%	82%	81%	84%	84%	78%	78%
Age	68	67	68	66	66	63	68	71	65	67	70	70.3	70.8
≥cT3	‐	‐	11%	‐	‐	‐	14%	20%	‐	14%	14%	30%	29%
Neoadj. chemo	0%	0%	14%	6%	0%	0%	15%	29%	0%	36%	27%	34%	36%
Median follow‐up	43 mo	43 mo	26 mo	122 mo	31 mo	42 mo	120 mo		38 mo	36 mo^a^	36 mo^a^	50 mo	20 mo
Removed nodes	19	31	17	‐	‐	27	38	41	18	26	23	12	16
Positive surgical margins	8.9%	8.6%	‐	‐	‐	‐	7.2%	7.8%	‐	5%	6%	7%	0%
pN+	28%	22%	20%	23%	24%	19%	22%	22%	18%	23%	16%	22%	12%^b^ (18%)
≥pT3	49%	40%	37%	29%	52%^c^	29%	20%	17%	33%	31%	32%	39%	32%
5‐yr RFS	59.2%	64.6%	66%	68%	62%	69%	65%	66%	69.5%			63%	
5‐yr OS	49.7%	58.9%	60%	66%	59%	68%	55%	62%	57.9%			57%	
3‐yr RFS	≈65%	≈73%	70%	≈72%	≈67%	≈73%	67%	66%	≈73%	65.4%	68.4%	67%	86%
3‐yr OS	≈60%	≈68%	69%	≈67%	≈65%	≈73%	62%	65%	≈67%	68.5%	73.9%	69%	90%

*Note*: No: number. cT: clinical stage (preoperative staging). pN+: patients with lymph node metastases in final pathology report. ‐: data not found in the articles presented. RFS: recurrence free survival. OS: overall survival. ≈Estimated values from Kaplan–Meier curves in the articles.

^a^ Median follow up data not presented in the article. Presenting 3‐year calculated RFS and OS. pT: final histopathological stage.

^b^ Amount of lymph node metastases found after the pathologists learning curve.

^c^ TNM‐stage. According to 1997 International Union Against Cancer and the American Joint Committee on Cancer.

The specimens after EbRC and stdRC are formalin fixed postoperatively. The stdRC lymph node specimen is dissected, extracted, and sent for evaluation in separate containers. Identifying lymph nodes in the formalin‐fixed en bloc RC specimen was a challenging procedure for the pathologists, but after 1 year of experience with EbRC, the pathologists were able to find the same number of lymph nodes in the EbRC specimen as in the stdRC specimen. When dividing the EbRC patients into three equal temporal groups (*n* = 48/49/48) according to date of surgery, the pathologist found 6% lymph node metastases in the first group, 10% in the second group, and 19% in the third group. The first group had the highest percentage of stages ≥pT3 (40%/31%/27%). There is no significant difference in the presence of lymph node metastases in the third group compared with the stdRC group (19% vs. 22%, *p* = 0.3). This represents the pathologists' learning curve for evaluating the EbRC specimens. Increasing administration of neoadjuvant chemotherapy corresponds with increased rates of pT0N0.^7^ We found a 5% difference in neoadjuvant chemotherapy between the two groups. This also accounts for differences in pN+ and pT− stages.

Surgical skills develop over time, and the learning curve complicates the comparison of patients treated within different timeframes. In the majority of the cases, patients treated with stdRC were operated on with the more experienced surgeon present. Equal LR rates were found for both surgeons after stdRC. The less experienced surgeon completed 145/145 EbRC cases, with the more experienced surgeon present in 35% of those cases. If the results are biased by any learning curve, it is more likely to be to the disadvantage of the EbRC group.

This study clearly has its limitations in comparing two historical patient populations and the time of follow up. Comparing two groups of consecutively treated patients with an overlapping treatment period of 18 months is challenging. Differences between the two study groups regarding the use of robot‐assisted surgery, blood loss, lymph node dissection, preoperative N‐stage and CIS, stage in the final pathology report, pN+ stage, and positive margins were noted. Multivariable Cox regression analyses and analyses based on propensity scored–matched groups were conducted to address this issue. Differences in lymph node dissection and positive margins were not adjusted for, as it interferes with the treatment effect of the new technique. The significantly improved oncological outcomes of the EbRC group persisted in both statistical methods.

Four out of seven LRs in the EbRC group were found within 7 months of follow‐up. LR appeared early in the follow‐up after RC in both study populations (Figure 3A–D). Elsayed et al.^4^ reported an 8‐month median time to disease relapse (LR and distant metastases). Only few events appears later than 3 years after RC in Kaplan–Meier analyses presenting recurrence after RC.^4^, ^5^, ^6^

All referring and follow up hospitals in Mid‐Norway are connected to one patient journal system, and all clinicians have full access to all radiological examinations and tests executed in the region. Regional urological multidisciplinary team meetings are held weekly and were established in January 2012. Surveillance protocols at our centre have been unchanged since 2012. No cancer specific deaths or deaths by other causes were missed. All patients in both groups had the same preoperative work up and were considered for neoadjuvant chemotherapy (if cT ≥ 2). The chemotherapy regimens were unchanged throughout the study period. None of the patients in both groups were included in immunotherapy studies. Immunotherapy was only administered as second line therapy after recurrence was diagnosed. None of the two patients who received second line immunotherapy had significant response influencing our results. Only three patients were lost to follow up due to emigration leading to consistent and complete data capture.

Perioperative complications in both groups were similar to published rates in other series.^4^, ^6^ The lower rate of Clavien–Dindo grade 2 complications in the EbRC group is explained by lower blood transfusion rates after robot‐assisted surgery. Readmission rates and mortality rates at 90 days were similar in the two groups. No additional risks of complications nor side effects were found for patients treated with EbRC during follow up.

There is a strong biological and oncological rationale behind EbRC based on the similarities in the embryonic development of the venous and lymphatic drainage of both the bladder and rectum. These facts account for the similarity of risk factors for recurrence in both cancers.^12^ By adhering to the principles of *the circumferential resection margin* the results after implementing EbRC have shown improved oncological outcomes that mirror the historical data after implementing TME for rectal cancer.

## 5 CONCLUSION

En bloc radical cystectomy characterised by systematic uninterrupted mobilisation of the associated venous and lymphovascular tissue reduced local and distant recurrences and improved survival for patients with muscle invasive or high‐grade superficial bladder cancer.

## DISCLOSURE OF INTEREST

The authors have no conflicts of interest to declare.

## AUTHOR CONTRIBUTIONS

All authors made significant contributions to this work. Design and funding: EK and CJA. Data collection: EK. Statistical analyses: EK and ØSa. Writing the manuscript: EK, ØSa, SL, ØS, AW and CJA. EK (EbRC), EK and CJA (stdRC) performed all surgical procedures.

## Supporting information


**Supporting Information S1.** Kaplan‐Meier curves comparing **1a:** local recurrence‐free survival, **1b:** recurrence‐free survival, **1c:** cancer specific survival, and **1d:** overall survival, for patients with ≥cT2 in both groups. The number of patients followed without an event in each group are reported annually.

**Supporting Information S2.** Multivariable cox regression analyses

**Supporting Information S3.** Kaplan‐Meier curves comparing **3a:** local recurrence‐free survival, **3b:** recurrence‐free survival, **3c:** cancer specific survival, and **3d:** overall survival, in propensity score matched groups. The number of patients followed without an event in each group are reported annually.
