# Supplementary material for: En bloc radical cystectomy: An overview of the technique and oncological results
Source: BJUI Compass. 2022 Sep 18;4(2):195–205. doi: 10.1002/bco2.190 (PMC9931536; doi:10.1002/bco2.190)
Supplement: Supplementary file 1 — Supporting Information S1. Kaplan‐Meier curves comparing 1a: local recurrence‐free survival, 1b: recurrence‐free survival, 1c: cancer specific survival, and 1d: overall survival, for patients with ≥cT2 in both groups. The number of patients followed without an event in each group are reported annually. [file BCO2-4-195-s003.docx]

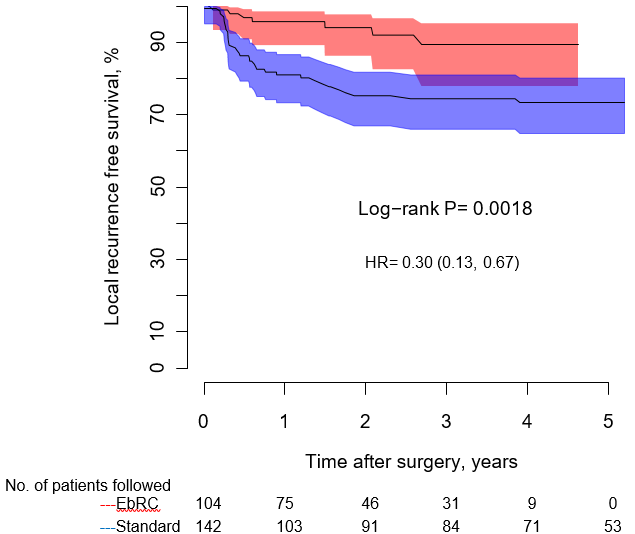

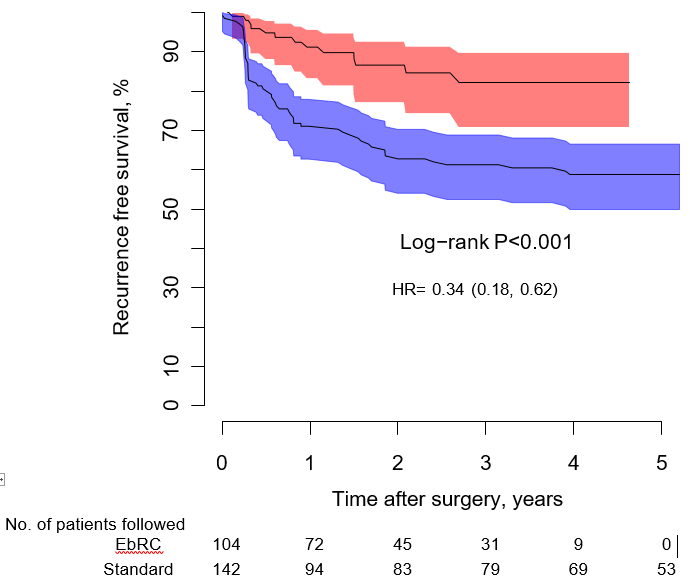


***Supplement: 1***

**B**

**A**


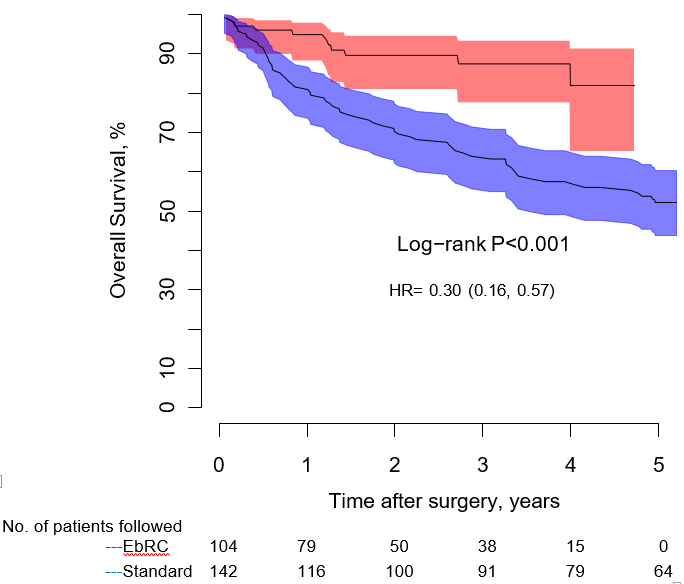

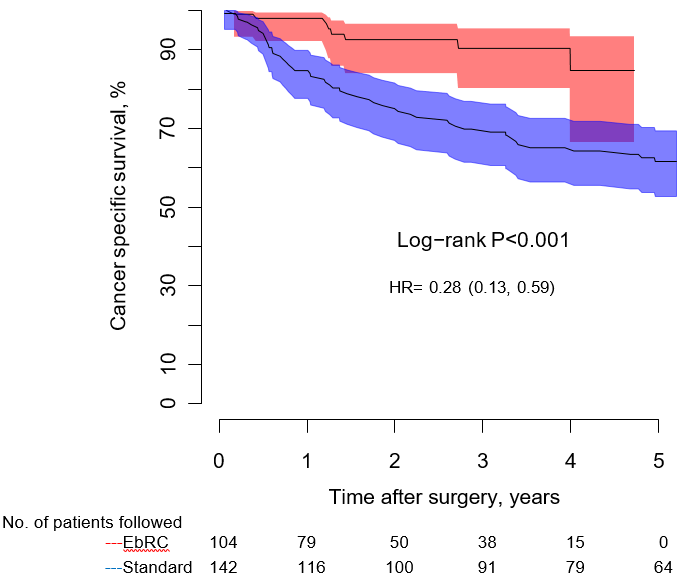


**D**

**C**

Kaplan-Meier curves comparing **1a:** local recurrence-free survival, **1b:** recurrence-free survival, **1c:** cancer specific survival, and **1d:** overall survival, for patients with ≥cT2 in both groups. *The number of patients followed without an event in each group are reported annually.*
