# Supplementary material for: En bloc radical cystectomy: An overview of the technique and oncological results
Source: BJUI Compass. 2022 Sep 18;4(2):195–205. doi: 10.1002/bco2.190 (PMC9931536; doi:10.1002/bco2.190)
Supplement: Supplementary file 2 — Supporting Information S2. Multivariable cox regression analyses [file BCO2-4-195-s001.docx]

| Multivariable Cox regression analyses: | |  |  |
| --- | --- | --- | --- |
| Local recurrence free survival: |  |  |  |
|  | HR: | 95% CI | p= |
| Unadjusted EbRC vs STDRC | 0.26 | 0.12 – 0.59 | ≤0.001 |
|  |  |  |  |
| Adjusted EbRC vs STDRC | 0.29 | 0.13 – 0.65 | 0.003 |
|  |  |  |  |
| Age | 0.98 | 0.93 – 1.03 | 0.33 |
| Gender | 2.02 | 1.05 – 3.90 | 0.036 |
| Neoadjuvant chemotherapy | 0.78 | 0.33 – 1.83 | 0.6 |
| CCI | 1.43 | 1.03 – 1.99 | 0.03 |
| cN | 2.61 | 1.17 – 5.83 | 0.02 |
| CIS | 0.93 | 0.50 – 1.75 | 0.8 |
| pT0-1 | - | - | - |
| pT2 | 4.18 | 0.85 – 20 | 0.079 |
| pT3-4 | 13.0 | 2.13 – 80 | 0.006 |
| pTNM 0-1 | - | - | - |
| pTNM 2 | 1.35 | 0.11 – 17 | 0.8 |
| pTNM 3-4 | 1.49 | 0.10 – 22 | 0.8 |
|  |  |  |  |
| Recurrence free survival : |  |  |  |
|  | HR: | 95% CI | p= |
| Unadjusted EbRC vs STDRC | 0.32 | 0.18 – 0.58 | ≤0.001 |
|  |  |  |  |
| Adjusted EbRC vs STDRC | 0.36 | 0.2 – 0.64 | ≤0.001 |
|  |  |  |  |
| Age | 0.98 | 0.94 – 1.01 | 0.21 |
| Gender | 1.69 | 1.02 – 2.81 | 0.043 |
| Neoadjuvant chemotherapy | 0.74 | 0.40 – 1.38 | 0.3 |
| CCI | 1.31 | 1.02 – 1.69 | 0.037 |
| cN | 2.65 | 1.45 – 4.83 | 0.002 |
| CIS | 1.02 | 0.64 – 1.65 | 0.91 |
| pT0-1 | - | - | - |
| pT2 | 2.01 | 0.82 – 4.95 | 0.13 |
| pT3-4 | 4.15 | 1.52 – 11 | 0.006 |
| pTNM 0-1 | - | - | - |
| pTNM 2 | 1.44 | 0.39 – 5.31 | 0.59 |
| pTNM 3-4 | 2.19 | 0.53 – 9.09 | 0.28 |
|  |  |  |  |
|  |  |  |  |
|  |  |  |  |
| Cancer specific survival : |  |  |  |
|  | HR: | 95% CI | p= |
| Unadjusted EbRC vs STDRC | 0.25 | 0.12 – 0.53 | ≤0.001 |
|  |  |  |  |
| Adjusted EbRC vs STDRC | 0.28 | 0.13 – 0.60 | 0.001 |
|  |  |  |  |
| Age | 0.97 | 0.94 – 1.02 | 0.22 |
| Gender | 1.72 | 1.00 – 2.97 | 0.051 |
| Neoadjuvant chemotherapy | 0.77 | 0.39 – 1.51 | 0.4 |
| CCI | 1.29 | 0.98 – 1.71 | 0.071 |
| cN | 2.59 | 1.36 – 4.92 | 0.004 |
| CIS | 0.88 | 0.51 – 1.51 | 0.6 |
| pT0-1 | - | - | - |
| pT2 | 2.16 | 0.78 – 6.0 | 0.14 |
| pT3-4 | 4.66 | 1.55 – 14 | 0.006 |
| pTNM 0-1 | - | - | - |
| pTNM 2 | 1.88 | 0.34 – 10 | 0.5 |
| pTNM 3-4 | 3.22 | 0.54 – 19 | 0.2 |
|  |  |  |  |
|  |  |  |  |
|  |  |  |  |
|  |  |  |  |
| Overall survival |  |  |  |
|  | HR: | 95% CI | p= |
| Unadjusted EbRC vs STDRC | 0.28 | 0.15 – 0.52 | ≤0.001 |
|  |  |  |  |
| Adjusted EbRC vs STDRC | 0.30 | 0.16 – 0.57 | ≤0.001 |
|  |  |  |  |
| Age | 0.99 | 0.96 – 1.02 | 0.6 |
| Gender | 1.32 | 0.82 – 2.14 | 0.3 |
| Neoadjuvant chemotherapy | 0.85 | 0.47 – 1.51 | 0.6 |
| CCI | 1.30 | 1.04 – 1.64 | 0.019 |
| cN | 1.99 | 1.13 – 3.51 | 0.018 |
| CIS | 1.25 | 0.82 – 1.93 | 0.3 |
| pT0-1 | - | - | - |
| pT2 | 2.24 | 1.00 – 5.02 | 0.049 |
| pT3-4 | 3.91 | 1.56 – 9.82 | 0.004 |
| pTNM 0-1 | - | - | - |
| pTNM 2 | 1.02 | 0.35 – 3.0 | 1 |
| pTNM 3-4 | 1.49 | 0.45 – 4.92 | 0.5 |

Supplement 2: Multivariable cox regression analyses Multivariable cox regression analysis performed for local recurrence free survival , recurrence free survival, cancer specific survival and overall survival adjusting for age, gender, neoadjuvant chemotherapy, CCI: Charlson comorbidity Index, cN: lymph node metastases at diagnoses , CIS: carcinoma in situ, pT: pathological stage (final pathology report) and pTNM-stage (Union for International Cancer Control, eight edition). EbRC: en-bloc radical cystectomy. STDRC: standard radical cystectomy. HR: Hazard ratio. CI: Confidence interval.
