# Supplementary material for: En bloc radical cystectomy: An overview of the technique and oncological results
Source: BJUI Compass. 2022 Sep 18;4(2):195–205. doi: 10.1002/bco2.190 (PMC9931536; doi:10.1002/bco2.190)
Supplement: Supplementary file 3 — Supporting Information S3. Kaplan‐Meier curves comparing 3a: local recurrence‐free survival, 3b: recurrence‐free survival, 3c: cancer specific survival, and 3d: overall survival, in propensity score matched groups. The number of patients followed without an event in each group are reported annually. [file BCO2-4-195-s002.docx]

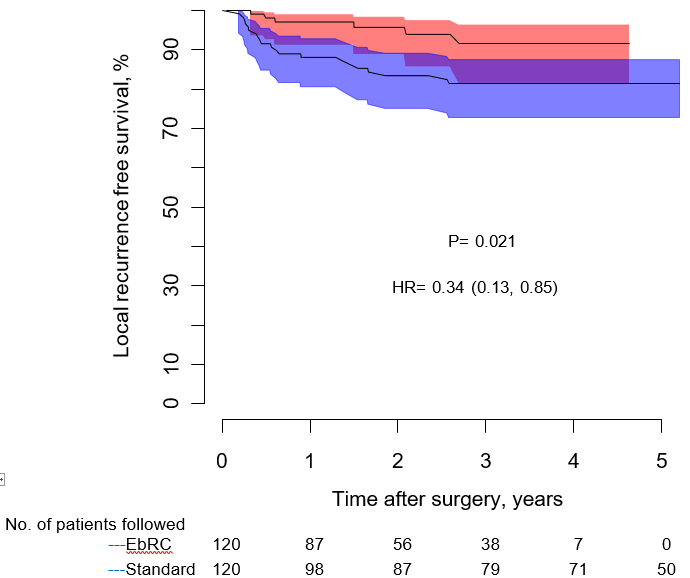

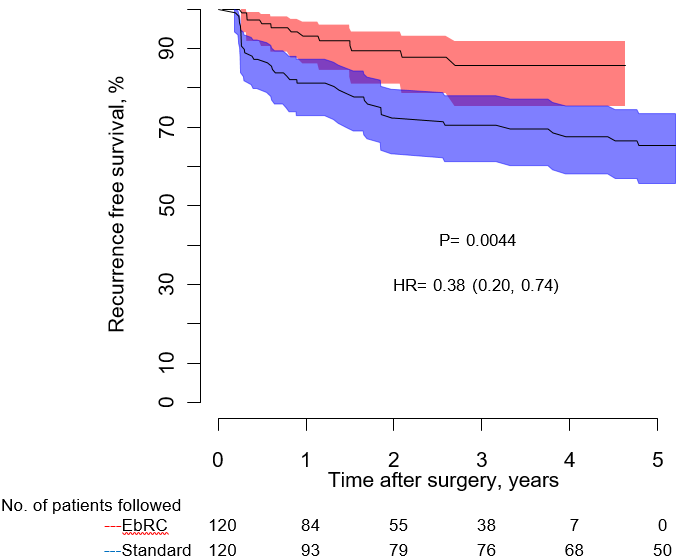


***Supplement: 3***

**A**

**B**


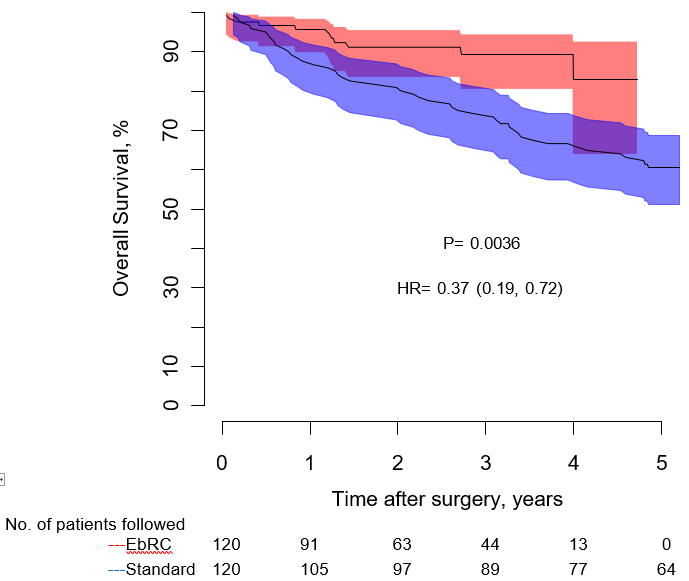


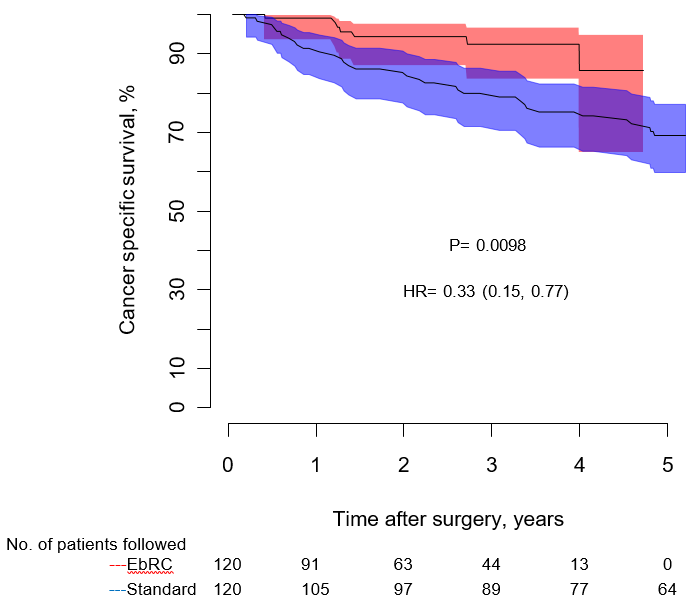


**D**

**C**

*Kaplan-Meier curves comparing* ***3a:*** *local recurrence-free survival,* ***3b:*** *recurrence-free survival,* ***3c:*** *cancer specific survival, and* ***3d:*** *overall survival, in propensity score matched groups. The number of patients followed without an event in each group are reported annually.*
